# Gene expression microarray data from mouse CBS treated with rTMS for 30 days, mouse cerebrum and CBS treated with rTMS for 40 days

**DOI:** 10.1016/j.dib.2018.01.079

**Published:** 2018-02-08

**Authors:** Tetsurou Ikeda, Satoru Kobayashi, Chikao Morimoto

**Affiliations:** a Laboratory for Structural Neuropathology, RIKEN Brain Science Institute, 2-1 Hirosawa, Wako City, Saitama 351-0198, Japan; b Clinical Immunology, Institute of Medical Science, The University of Tokyo, 4-6-1 Shirokanedai, Minato-ku, Tokyo 108-8639, Japan; c Technical Support, Thermo Fisher Scientific K.K., 3-9 Moriya, Kanagawa-ku, Yokohama 221-0022, Japan

**Keywords:** CBS, cerebellum with brain stem, rTMS, Microarray data

## Abstract

This data article contains complementary tables related to the research article study entitled, ‘Effects of repetitive transcranial magnetic stimulation on ER stress−related genes and glutamate, γ−aminobutyric acid, and glycine transporter genes in mouse brain’ (Ikeda et al. (2017) [1]), which showed that rTMS modulates glutamate, GABA and glycine transporters and regulates ER stress−related genes. Here, we provide accompanying data collected using Affymetrix GeneChip microarrays to identify changes in gene expression in mouse CBS treated with rTMS for 30 days (Tables 1–21) and mouse cerebrum (Tables 22–57) and CBS (Tables 58–94) treated with rTMS for 40 days.

**Specifications Table**

**Table:** 

Subject area	*Neuroscience*
More specific subject area	*Gene expression*
Type of data	*Tables*
How data was acquired	*Affymetrix GeneChip RNA microarray*
Data format	*Filtered, analysed*
Experimental factors	*Mouse brain treated with rTMS for 30 and 40 days*
Experimental features	*RNA isolation, global gene expression analyses*
Data source location	*Wako, Saitama, Japan*
Data accessibility	*Data are contained within this article*

**Value of the data**

• Global gene expression analysis of mouse cerebellum with brain stem (CBS) treated with repetitive transcranial magnetic stimulation (rTMS) for 30 and 40 days
• These data may be useful for comparison with microarray data obtained from rTMS of different durations.
• Genes identified as differentially expressed in this data set could be useful in further studies investigating the effects of rTMS on mouse brain.
• In contrast to the advantage of using microelectrodes, inflammation never occurs while using TMS because it is non−invasive. Immune system may recognise the microelectrode and cause inflammation.

## 1 Data

Affymetrix GeneChip microarray analyses of mRNA isolated from mouse CBS after 30days rTMS showed altered expression of several genes (Tables 1–21), including glutamatergic, GABAergic and glycinergic (e.g. glycine transporter) neurotransmission systems and ER stress−related genes. Affymetrix GeneChip microarray analyses of mRNA isolated from mouse cerebrum after 40 days rTMS also showed altered expression of several genes (Tables 22–57), including glutamatergic (e.g. glutamate transporters), GABAergic and glycinergic neurotransmission systems. Furthermore, Affymetrix GeneChip microarray analyses of mRNA isolated from mouse CBS after 40days rTMS showed altered expressions of several genes (Tables 58–94), including glutamatergic (e.g. glutamate transporters), GABAergic and glycinergic neurotransmission systems. Mice CBS and cerebrum stimulated by rTMS for 30 or 40days were denoted as M1 and M2, and sham control were denoted as C1 and C2 (n=2). All the expression ratios were converted into the log_2_ (expression ratio) values. L1: Signal Log Ratio (M1/C1), L2: Signal Log Ratio (M2/C1), L3: Signal Log Ratio (M1/C2), L4: Signal Log Ratio (M1/C2). C1 and C2 were used as control, and M1 and M2 were normalized with regard to C1 and C2 to obtain expression ratios. Expression Console (EC) Ver.1.4 and Transcriptome Analysis Console (TAC) Ver.3 were used for comparison analysis as a manufacturer procedure. C means data analysis output for a comparison analysis showing change p−values with the associated Increase (I) or Decrease (D) call. Increase calls have change p−values closer to zero and Decrease calls have Change p−values closer to one. Finally, the change algorithm assesses probe pair saturation, calculates a change p−values, and assigns an (I), marginal increase (MI), no change (NC), marginal decrease (MD), or (D) call for C. Genes with more than two significant difference calls were chosen. Abbreviations; TC ID: Transcript Cluster ID, GS: gene symbol, *: Total number of increase, #: Total number of decrease. TC ID is available for pathway analysis.

## 2 Experimental design, materials and methods

We performed a comprehensive analysis of altered gene expression in CBS after chronic rTMS by using a high−density oligonucleotide array (GeneChip; Affymetrix, Santa Clara, CA, USA. MG_U74Av2 probe array), as described elsewhere [2]. Using the Affymetrix algorithm [3] and multiple analysis comparison software for assessing gene expression differences, mRNAs that increased or decreased in the mouse brain after chronic rTMS relative to levels in the control mouse brain were identified. Pathway analysis was used to identify the significant pathway of the differential genes based on KEGG. Furthermore, gene ontology (GO) analysis was performed to analyse the main function of the differentially expressed genes based on GO, which is the key functional classification of NCBI that can organise genes into hierarchical categories and uncover the gene regulatory network based on biological process and molecular function [4]. Stimulation was performed using a round−coil (7.5 cm outer diameter) and a Nihon Kohden Rapid Rate Stimulator (Nihon Kohden, Japan). Stimulation conditions were as follows: 20 Hz, 2 s; 20 times/day; inter−stimulus 1−min interval (30% machine output, representing approximately 0.75 T). The coil was placed over the head without touching the skull. Sham control mice were ‘stimulated’ >10 cm from the head. rTMS did not produce either notable seizures or changes in behaviour, such as excessive struggling. The animals were killed 24 h after the last stimulation, and their brains were processed for further analysis [6], [7]. Whole mouse brain was divided at the midbrain into the cerebrum and CBS. This method of stimulation is applied to the whole brain but not to specific regions of brain. Hence, the feedback effect of the afferent pathway should be considered. Total RNA was isolated from the cerebrum and CBS by acid–phenol extraction [5]. Poly(A)+ RNA was isolated from the samples using an mRNA purification kit (TaKaRa Bio, Japan).
