# Supplementary material for: Gene expression microarray data from mouse CBS treated with rTMS for 30 days, mouse cerebrum and CBS treated with rTMS for 40 days
Source: Data Brief. 2018 Feb 8;17:1078–81. doi: 10.1016/j.dib.2018.01.079 (PMC5988379; doi:10.1016/j.dib.2018.01.079)
Supplement: Supplementary file 2 — Supplementary material. [file mmc2.docx]

**Table1. Gene expression matrix after 30 days rTMS on CBS.**

Ndufb9, Apoe, Cdk5, Cyc1, Uqcrc2 and Uqcr10 are genes related to Alzheimer's disease. Ndufb9, Cdk5, Cyc1, Uqcrc2 and Uqcr10 are genes related to Parkinson's disease and Huntington's disease. Ndufb9, Ckmt1, Cyc1, Ptgds, Uqcrc2 and Uqcr10 are genes related to Metabolic pathways. Use1 and Vamp1 are genes related to SNARE interactions in vesicular transport.

**Table2. Gene expression matrix after 30 days rTMS on CBS.**

Mrpl12, Rpl18, Rpl37 and Rplp0 are genes related to Ribosome.

**Table3. Gene expression matrix after 30 days rTMS on CBS.**

Ndufa7, Ndufb2, Ndufb7, Cox5b, Gm4943 and Uchl1 are genes related to Parkinson's disease, Alzheimer's disease and Huntington's disease.Ndufa7, Ndufb2, Ndufb7, Cmbl, Ckb, Cox5b, Dctpp1,Pfkm, Pgk1,Psat1, Gm4943 are genes related to Metabolic pathways.

**Table4. Gene expression matrix after 30 days rTMS on CBS.**

No genes were found in the specific pathway.

**Table5. Gene expression matrix after 30 days rTMS on CBS.**

Epas1, Igf1r, Jup, Crkl and Vegfa are genes related to Pathways in cancer. Gabrb2, Gabra2, Grid2 and Glra1 are genes related toNeuroactive ligand-receptor interaction.

**Table 6. Gene expression matrix after 30 days rTMS on CBS.**

Atp2b2, Atp1b2, Fos, Rapgef4, Gria2 and Pik3r1are genes related to cAMP signaling pathway. Cacna1a, Gria2, Itpr1 and Kif5care genes related to Dopaminergic synapse. Fos, Gria2, Itpr1 and Per1 are genes related to Circadian entrainment. Cacna1a, Gria2, Itpr1 and Trpc1 are genes related to Glutamatergic synapse.

**Table 7. Gene expression matrix after 30 days rTMS on CBS.**

Vamp2 and Vamp3 are genes related to SNARE interactions in vesicular transport.

**Table 8. Gene expression matrix after 30 days rTMS on CBS.**

Pten, Ddit4, Irs2 are genes related toMicroRNAs in cancer.

**Table 9. Gene expression matrix after 30 days rTMS on CBS.**

Gabra6, Gad2 and Slc38a2 are genes related toGABAergic synapse.

**Table 10. Gene expression matrix after 30 days rTMS on CBS.**

Gabrb3, Kcnj9 and Kcnd2 are genes related to Serotonergic synapse. Gria3,Kcnj9 and Ppp1cb are genes related to Dopaminergic synapse.

**Table 11. Gene expression matrix after 30 days rTMS on CBS.**

Gria4, Gnao1, Itpr2 and Th are genes related to Dopaminergic synapse. Gria4, Gnao1 and Itpr2 are genes related to Glutamatergic synapse.

**Table 12. Gene expression matrix after 30 days rTMS on CBS.**

Rad52 and Pold1 are genes related to Homologous recombination.Mbd4 and Pold1 are genes related to Base excision repair.

**Table 13. Gene expression matrix after 30 days rTMS on CBS.**

Hbb-b2 and Thop1 are genes related to African trypanosomiasis.

**Table 14. Gene expression matrix after 30 days rTMS on CBS.**

Rab10, Pdgfra and Smap1 are genes related to Endocytosis.

**Table 15. Gene expression matrix after 30 days rTMS on CBS.**

|  |  |
| --- | --- |
|  |  |

PAXBP1,Arrb1,Nr5a1,Baz1b,Rprd1b,Stat5b,Tenm2,Txnip and Zfpm2 are genes related to Endocytosis.

**Table 16. Gene expression matrix after 30 days rTMS on CBS.**

Atp6v1b2 and Car2 are genes related to Collecting duct acid secretion. Cebpb, Prom1 and Runx1t1 are genes related to Transcriptional misregulation in cancer.

**Table 17. Gene expression matrix after 30 days rTMS on CBS.**

Atp1a1, Cacng2 and Camk2a are genes related to Adrenergic signaling in cardiomyocytes.

**Table 18. Gene expression matrix after 30 days rTMS on CBS.**

Glul, Pld2 and Ppp3ca are genes related to Glutamatergic synapse. Slc6a9 are genes related to Glycine synapse.

**Table 19. Gene expression matrix after 30 days rTMS on CBS.**

Frmd6, Csnk1d, Prkcz and Tcf7l2 are genes related toHippo signaling pathway.Csnk1d and Cry2 are genes related toCircadian rhythm.

**Table 20. Gene expression matrix after 30 days rTMS on CBS.**

Bcl2, Tbk1 and Nfatc3 are genes related to Hepatitis B.

**Table 21. Gene expression matrix after 30 days rTMS on CBS.**

Bmpr2, Cxcr5, Csf2ra, Kit and Kitl are genes related to Cytokine-cytokine receptor interaction.

**Table 22. Gene expression matrix after 40 days rTMS on cerebrum.**

d2 and Kdm6a are genes related to Transcriptional misregulation in cancer.

**Table 23. Gene expression matrix after 40 days rTMS on cerebrum.**

Atp1b3 and Car4 are genes related to Proximal tubule bicarbonate reclamation.

**Table 24. Gene expression matrix after 40 days rTMS on cerebrum.**

Cdkn1b, Fgf1, Fgf9, Lpar1 and Reln are genes related to PI3K-Akt signaling pathway.

**Table 25. Gene expression matrix after 40 days rTMS on cerebrum.**

Gria4 is a gene related to Glutamatergic synapse.

**Table 26. Gene expression matrix after 40 days rTMS on cerebrum.**

Rora and Arntl are genes related to Circadian entrainment.

**Table 27. Gene expression matrix after 40 days rTMS on cerebrum.**

Car4 and Car4 are genes related to Nitrogen metabolism.

**Table 28. Gene expression matrix after 40 days rTMS on cerebrum.**

Itgb4, Pxn, Pip5k1b and Crkl are genes related to Regulation of actin cytoskeleton.

**Table 29. Gene expression matrix after 40 days rTMS on cerebrum.**

Abca2, Ap4s1 and Idua are genes related to Lysosome.

**Table 30. Gene expression matrix after 40 days rTMS on cerebrum.**

Tardbp、Atl2, Cnn3, Hspb8, Mybpc3 and Rhoq are genes related to methylation.

**Table 31. Gene expression matrix after 40 days rTMS on cerebrum.**

Ctbp2 and Cdkn1b are genes related to Chronic myeloid leukemia.

**Table 32. Gene expression matrix after 40 days rTMS on cerebrum.**

Agap1 and Fdx1 are genes related to adrenal grand.

**Table 33. Gene expression matrix after 40 days rTMS on cerebrum.**

Nkx2-2 and Neurod1are genes related tomaturity onset diabetes of the young.

**Table 34. Gene expression matrix after 40 days rTMS on cerebrum.**

Atp2a3, Nae1 and Itpr1 are genes related to Alzheimer's disease.

**Table 35. Gene expression matrix after 40 days rTMS on cerebrum.**

Cacng2, Clic4, Hcn2 and Slc38a2 are genes related to ion transport.

**Table 36. Gene expression matrix after 40 days rTMS on cerebrum.**

Mff, Mfn1 and Rmdn3 are genes related to mitochondrion outer membrane.

**Table 37. Gene expression matrix after 40 days rTMS on cerebrum.**

Atp6v1g1, Auh, St6 galnac4, Mtm1, Aldh9a1 and Sat1 are genes related to metabolic pathways.

**Table 38. Gene expression matrix after 40 days rTMS on cerebrum.**

Hibadh, Dld and Hadhb are genes related to Valine, leucine and isoleucine degradation.

**Table 39. Gene expression matrix after 40 days rTMS on cerebrum.**

Calb1, Cltb and Pth1r are genes related to Endocrine and other factor-regulated calcium reabsorption.

**Table 40. Gene expression matrix after 40 days rTMS on cerebrum.**

Fam21, Llgl1, Rnf8 and Tmpo are genes related to embryo.

**Table 41. Gene expression matrix after 40 days rTMS on cerebrum.**

Ak4, Dguok, Hcn1 and Kit are genes related to nucleotide-binding.

**Table 42. Gene expression matrix after 40 days rTMS on cerebrum.**

Fos, Gnao1, Kif5a and Kif5c are genes related to Dopaminergic synapse. Gabra2, Gabrb1 and Gnao1 are genes related to GABAergic synapse. Fos, Gnao1 and Per1 are genes related to Circadian entrainment. Grik5, Gnao1 and Trpc1 are genes related to Glutamatergic synapse.

**Table 43. Gene expression matrix after 40 days rTMS on cerebrum.**

Egr1 and Mapk1 are genes related to Prion diseases. Cx3cl1, Mapk1, Rhoa are genes related to Chemokine signaling pathway.

**Table 44. Gene expression matrix after 40 days rTMS on cerebrum.**

Camk2b, Gng2, Mapk10 and Th are genes related to Dopaminergic synapse.

**Table 45. Gene expression matrix after 40 days rTMS on cerebrum.**

Adrb1 and Chrm1 are genes related to calcium signaling pathway.

**Table 46. Gene expression matrix after 40 days rTMS on cerebrum.**

Acvr2a, Pik3r2 and Wnt4 are genes related to Signaling pathways regulating pluripotency of stem cell.

**Table 47. Gene expression matrix after 40 days rTMS on cerebrum.**

Smad2, Csf1r, Epn1, Nedd4l and Pip5k1a are genes related to Endocytosis.

**Table 48. Gene expression matrix after 40 days rTMS on cerebrum.**

Grin1, Homer2 and Slc1a1 are genes related to Glutamatergic synapse.

**Table 49. Gene expression matrix after 40 days rTMS on cerebrum.**

Ctnna2, Mapk10, Rxrg, Tgfa and Zbtb16 are genes related to Pathways in cancer.

**Table 50. Gene expression matrix after 40 days rTMS on cerebrum.**

Gfra2, Plcb1 and Ppp2r2b are genes related to Dopaminergic synapse. Rapgef4, Plcb1 and Ppp2r2b are genes related to Adrenergic signaling in cardiomyocytes.

**Table 51. Gene expression matrix after 40 days rTMS on cerebrum.**

Camk2a, Mapk1 and Ppp2r5a are genes related to Adrenergic signaling in cardiomyocytes.

**Table 52. Gene expression matrix after 40 days rTMS on cerebrum.**

Atp6v1e1, Tubb2a and Tubb5 are genes related to Phagosome.

**Table 53. Gene expression matrix after 40 days rTMS on cerebrum.**

Gldc and Grhpr are genes related toGlycine, serine and threonine metabolism.

**Table 54. Gene expression matrix after 40 days rTMS on cerebrum.**

Brk1, Git1, Ptk2 and Tmsb4x are genes related to Regulation of actin cytoskeleton.

**Table 55. Gene expression matrix after 40 days rTMS on cerebrum.**

Drd2、Sstr2　and Trhr are genes related to Neuroactive ligand-receptor interaction.

**Table 56. Gene expression matrix after 40 days rTMS on cerebrum.**

Gng10, Kif5a, Prkcb and Ppp2r5care genes related to Dopaminergic synapse.

**Table 57. Gene expression matrix after 40 days rTMS on cerebrum.**

Gabrb3 and Gng4 are genes related to GABAergic synapse.

**Table 58. Gene expression matrix after 40 days rTMS on CBS.**

Arhgdig and Mapk3 are genes related toNeurotrophin signaling pathway.Npy and Mapk3 are genes related toAlcoholism.

**Table 59. Gene expression matrix after 40 days rTMS on CBS.**

Gria2, Prkcb and Gng2 are genes related to Glutamatergic synapse.

**Table 60. Gene expression matrix after 40 days rTMS on CBS.**

Psme1 and Psmb10 are genes related to Proteasome.

**Table 61. Gene expression matrix after 40 days rTMS on CBS.**

Adcy2, Rgs14, Apbb1ip, asgrp2 and Tln1 are genes related to Rap1 signaling pathway.

**Table 62. Gene expression matrix after 40 days rTMS on CBS.**

Fosb and Dlg4 are genes related to Cocaine addiction.

**Table 63. Gene expression matrix after 40 days rTMS on CBS.**

Grin1 and Slc1a1 are genes related to Glutamatergic synapse.

**Table 64. Gene expression matrix after 40 days rTMS on CBS.**

Epha5, Pak3 and Efnb2 are genes related to Axon guidance.

**Table 65. Gene expression matrix after 40 days rTMS on CBS.**

C1qc and Map2k1 are genes related to Prion diseases.

**Table 66. Gene expression matrix after 40 days rTMS on CBS.**

Gabarapl1, Gad2 and Prkacb are genes related to GABAergic synapse.

**Table 67. Gene expression matrix after 40 days rTMS on CBS.**

Ccnd1, Camk2a and Nlk are genes related to Wnt signaling pathway.

**Table 68. Gene expression matrix after 40 days rTMS on CBS.**

Cdkn1a, Ppp3ca and Oxt are genes related to Oxytocin signaling pathway.

**Table 69. Gene expression matrix after 40 days rTMS on CBS.**

Arhgdia, Calm1 and Maged1 are genes related to Neurotrophin signaling pathway.

**Table 70. Gene expression matrix after 40 days rTMS on CBS.**

Drd2, Gria3, Gnb1 and Ppp3cb are genes related to Dopaminergic synapse.

**Table 71. Gene expression matrix after 40 days rTMS on CBS.**

Cacna2d3, Cacnb3, Myh7 and Ppp2r5c are genes related to Adrenergic signaling in cardiomyocytes.

**Table 72. Gene expression matrix after 40 days rTMS on CBS.**

Gnai2, Prkcb and Ppp3r1 are genes related to Glutamatergic synapse.

**Table 73. Gene expression matrix after 40 days rTMS on CBS.**

Cacna1g, Grin1 and Per1 are genes related to Circadian entrainment.

**Table 74. Gene expression matrix after 40 days rTMS on CBS.**

Col1a1, Fn1 and Vcl are genes related to Amoebiasis. Col1a1, Fn1 and Reln are genes related to ECM-receptor interaction.

**Table 75. Gene expression matrix after 40 days rTMS on CBS.**

Fbxw11 and Csnk1d are genes related to Hedgehog signaling pathway.

**Table 76. Gene expression matrix after 40 days rTMS on CBS.**

Fyn and Stip1 are genes related to Prion diseases.

**Table 77. Gene expression matrix after 40 days rTMS on CBS.**

Ddx46, Hnrnpa3 and Srsf3 are genes related to Spliceosome.

**Table 78. Gene expression matrix after 40 days rTMS on CBS.**

Birc2, Itgb4 and Pxn are genes related to Focal adhesion.

**Table 79. Gene expression matrix after 40 days rTMS on CBS.**

Vwf, Crkl and Vegfa are genes related to Focal adhesion.

**Table 80. Gene expression matrix after 40 days rTMS on CBS.**

Atp2a3, Commd9, Heph, Lrrc8a and Slc9a8 are genes related to ion transport.

**Table 81. Gene expression matrix after 40 days rTMS on CBS.**

Apip and Tst are genes related toCysteine and methionine metabolism.

**Table 82. Gene expression matrix after 40 days rTMS on CBS.**

Gab1,Msn and Tim3 are genes related to Proteoglycans in cancer.

**Table 83. Gene expression matrix after 40 days rTMS on CBS.**

Mapk12, Stat5b and Sos2 are genes related to Prolactin signaling pathway.

**Table 84. Gene expression matrix after 40 days rTMS on CBS.**

Nefm and Plp1 are genes related to axon development.

**Table 85. Gene expression matrix after 40 days rTMS on CBS.**

Gabra1 and Gabra6 are genes related to Nicotine addiction.

**Table 86. Gene expression matrix after 40 days rTMS on CBS.**

Atp1b2, Asgr1 and Itpr1 are genes related to Thyroid hormone synthesis.

**Table 87. Gene expression matrix after 40 days rTMS on CBS.**

Gng11, Slc5a1, Trpc1 are genes related to Glutamatergic synapse. Elovl2 and Scd1 are genes related to Biosynthesis of unsaturated fatty acids.

**Table 88. Gene expression matrix after 40 days rTMS on CBS.**

Flt3, Itpr1, Igf2r and Myt1 are genes related to post-embryonic development.

**Table 89. Gene expression matrix after 40 days rTMS on CBS.**

Aldh9a1, Bckdha, Etfa, Hsdl2 and Sesn1 are genes related to oxidation-reduction process.

**Table 90. Gene expression matrix after 40 days rTMS on CBS.**

Ehd1, Tbc1d1 and Snx2are genes related to intracellular protein transport.

**Table 91. Gene expression matrix after 40 days rTMS on CBS.**

Dusp16, Fgfr1, Gna12 and Rps6ka1are genes related to MAPK signaling pathway.

**Table 92. Gene expression matrix after 40 days rTMS on CBS.**

Ank3 and Kcnd2are genes related to neuronal action potential.

**Table 93. Gene expression matrix after 40 days rTMS on CBS.**

Grik2 and Kcna2 are genes related to neuronal action potential.Gabrd and Mecp2 are genes related to chemical synaptic transmission. Grik2 and Mecp2 are genes related to excitatory postsynaptic potential.

**Table 94. Gene expression matrix after 40 days rTMS on CBS.**

Atp1a2, Atp1b3 and Ap2b1are genes related toEndocrine and other factor-regulated calcium reabsorption. Atp1a2 and Atp1b3 are genes related toAdrenergic signaling in cardiomyocytes.
